# Recent development of temperature-responsive surfaces and their application for cell sheet engineering

**DOI:** 10.1093/rb/rbu011

**Published:** 2014-10-20

**Authors:** Zhonglan Tang, Teruo Okano

**Affiliations:** Institute of Advanced Biomedical Engineering and Science, TWIns, Tokyo Women’s Medical University, 8-1 Kawada-cho, Shinjuku-ku, Tokyo 162-8666, Japan

**Keywords:** poly(*N*-isoproplyacrylamide), temperature-responsive cell culture surface, cell sheet, cell sheet engineering

## Abstract

Cell sheet engineering, which fabricates sheet-like tissues without biodegradable scaffolds, has been proposed as a novel approach for tissue engineering. Cells have been cultured and proliferate to confluence on a temperature-responsive cell culture surface at 37°C. By decreasing temperature to 20°C, an intact cell sheet can be harvested from the culture surface without enzymatic treatment. This new approach enables cells to keep their cell–cell junction, cell surface proteins and extracellular matrix. Therefore, recovered cell sheet can be easily not only transplanted to host tissue, but also constructed a three-dimensional (3D) tissue by layering cell sheets. Moreover, cell sheet manipulation technology and bioreactor have been combined with the cell sheet technology to fabricate a complex and functional 3D tissue *in vitro*. So far, cell sheet technology has been applied in regenerative medicine for several tissues, and a number of clinical studies have been performed. In this review, recent advances in the preparation of temperature-responsive cell culture surface, the fabrication of organ-like tissue and the clinical application of cell sheet engineering are summarized and discussed.

## Introduction

Cell-based regenerative therapy has been developed rapidly as a most promising therapeutic treatment for impaired or defective tissues and organs [1–7]. As the first generation of cell-based therapy, the direct injection of cell suspensions has already been performed in various clinical trials [5–9]. However, due to the enzyme treatments, isolated cells are found to be difficult to locate and survive in host tissues [8, 9]. It is speculated that only several percent of cultured cells are transplanted to target tissues, which significantly reduces the expected therapeutic effects [9]. To resolve these problems, in 1993, Langer and Vacanti [10] proposed the tissue engineering approach as the second generation of cell-based therapy. Biodegradable porous polymer scaffolds, such as polyglycolic acid [11], collagen gel [12], fibrin gel [13, 14] and gelatin [15, 16], seeded with cultured cells, have been used to fabricate three-dimensional (3D) tissue-like grafts. So far, tissue engineering approach based on biodegradable materials has been performed in various clinical trials, including epidermis [17, 18], bone [19, 20], cartilage [20, 21], blood vessels [22, 23] and heart valves [24, 25]. In addition, decellularized scaffold as a new approach for tissue engineering has been developed to construct a whole-organ, which provide an acellular, naturally occurring 3D biologic scaffold for cell culture [26]. Several encouraging results have obtained in animal models and clinical study [27, 28]. However, transplantation of scaffold-based tissue often results in a pathological state of fibrosis due to the low cell dense of constructed tissue, necrosis due to lack of microcapillaries and strong inflammatory responses due to the biodegradation of scaffolds.

In contrast to scaffold-based tissue engineering, a scaffold-free tissue engineering methodology has developed by our laboratory [29, 30]. Cells have been cultured on an intelligent temperature-responsive cell culture surface. By reducing temperature from 37°C to 20°C, cultured cells can be harvested as an intact contiguous cell sheet, and transplanted to host tissue. This approach called as ‘cell sheet engineering’ has been used to fabricate tissue-like grafts with many kinds of cells and performed in several clinical trials successfully [31–35].

## Temperature-Responsive Cell Culture Surface for Cell Sheet Fabrication

**Figure 1.**
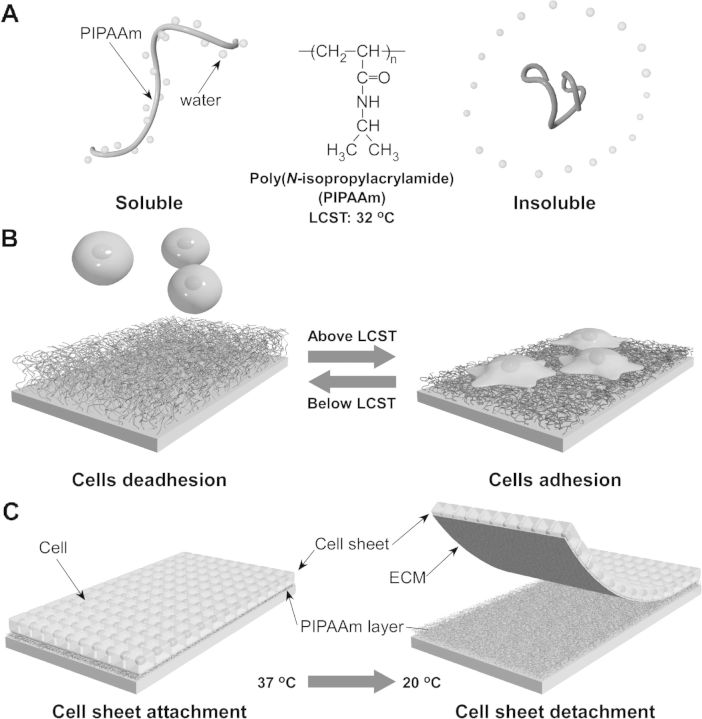
Schematic illustration of the temperature-responsive character change of PIPAAm in aqueous solution (**A**) and on substrate (**B**). Attaching cells can proliferate to confluence, and detach from PIPAAm-grafted substrate with ECM, when decreasing temperature from 37°C to 20°C (**C**).

Poly(*N*-isoproplyacrylamide) (PIPAAm) and its copolymer have been grafted on the surfaces of numerous materials to prepare the temperature-responsive cell culture surface. As a famous intelligent polymer, PIPAAm has reversible temperature-responsive soluble/insoluble character changes in aqueous solution below and above a lower critical solution temperature (LCST) of 32°C1 [36]. PIPAAm-grafted surface also shows reversible temperature-responsive characteristic depended on temperature change [29, 30, 33]. At 37°C, since the PIPAAm-grafted surface is hydrophobic, cells adhere and spread on the surface, and proliferate to confluence. By decreasing temperature to 20°C, due to the surface character change from hydrophobic to hydrophilic, cells detach from the surface spontaneously and form an intact cell sheet (Fig. 1). In 1990, Yamada *et al.* [29] and Okano *et al.* [30] have successfully grafted PIPAAm on commercially available tissue culture polystyrene (TCPS) dish by electron beam (EB) irradiation. At 37°C, primary hepatocytes attach to PIPAAm-grafted TCPS as well as to commercially TCPS reference. However, attaching hepatocytes can detach from the hydrated PIPAAm-grafted surface at 20°C without trypsin treatment or chelating agents. Cell recovery procedure with enzymatic treatment is found to impair cell membrane by cleaving various membrane-associated proteins and cutting off cell–cell junction, cell surface proteins and extracellular matrix (ECM), inducing the damage of cell function [37–39]. In contrast, further investigations demonstrate that cell sheet recovered from the PIPAAm-grafted TCPS may keep their membrane-associated proteins, which preserve cell function and allow cell sheet to adhere to host tissue [40, 41]. PIPAAm has been grafted onto TCPS covalently, and the thickness of PIPAAm layer plays the key factor to achieve cell adhesion/deadhesion on the surface [42, 43]. Cells adhesion/deadhesion in response to temperature changes has shown on the surface effectively with 15–20-nm thick PIPAAm layer, and cells barely adhere to the surface with more than 30-nm thick PIPAAm layer even at 37°C [42].

As effective approach especially for industrial manufacture, EB irradiation is usually used for modifying PIPAAm layer on the substrate. PIPAAm-grafted TCPS produced by EB irradiation is now commercially available, named as Nunc™ Dish with UpCell™ Surface, which can be purchased from Thermo Fisher Scientific Inc. However, EB irradiating equipment is expensive complicated machinery, which is hardly equipped in general laboratories. Several other approaches have been investigated as alternative preparation methods for PIPAAm modification (Table 1).

**Table 1.** Preparation of temperature-responsive cell culture surface

Modification procedure	Categories of the procedure
Irradiation method	Electron beam (EB) irradiation [29, 30, 42, 43]
	Plasma irradiation [44–48]
	Ultra violet (UV) irradiation [49–52]
Grafting polymer by ‘grafting-from’ method	Atom transfer radical polymerization (ATRP) [53–55]
	Reversible addition-fragmentation chain transfer radical polymerization (RAFT) [56]
Polymer coating method	Electrostatic coating approach [57–58]
	Physical coating approach [59–62]

### Irradiation method

Similar as EB irradiation, plasma and ultra violet (UV) irradiation are used as an alternative approach to prepare PIPAAm-grafted surface. For the plasma irradiation method, in a low vacuum and vaporized IPAAm-monomer atmosphere, high plasma power is employed to form an adhesion-promoting layer [44–46]. The layer is necessary to graft subsequent functional polymer deposition, which is carried out at successively reduced plasma power. Bovine aortic endothelial cell (BAEC) sheet has been harvested successfully from the resultant surface by reducing temperature from 37°C to 20°C. Principal component analysis of resultant surfaces after spontaneous cell sheet recovery by time-of-flight secondary ion mass spectrometry shows that laminin and bovine serum albumin components still remain on the surface [47, 48]. The immunoassay of PIPAAm-grafted surface shows that collagen component also remains on the surface, though the immunoassay of recovered cell sheet reveals that fibronectin (FN) component is associated with the cell sheet and hardly remains on the surface. The FN recovery is also in consistent with cell sheet recovered from PIPAAm-grafted TCPS prepared by the EB irradiation method. Significantly, PIPAAm-grafted surface prepared by plasma irradiation barely shows the PIPAAm thickness dependency on cell adhesion observed on PIPAAm-grafted TCPS by EB irradiation [46]. It is probably due to the polymer thickness variation of the deposited adhesion-promoting layer by the initial plasma polymerization.

Morra *et al.* have firstly used a UV irradiation method to graft PIPAAm on polystyrene (PS) dishes. PS dish containing IPAAm monomer and benzophenone (BP), a photoinitiator, dissolved in 2-propanol solution is subjected to UV irradiation for preparing PIPAAm gel-grafted surface [49]. Resultant surfaces can control L-929 mouse-fibroblast-cell attachment and detachment by changing temperature. Similarly, Ito *et al.* [50, 51] have developed a patterned PIPAAm-grafted surface by using poly(*N*-isopropylacrylamide-*co*-acrylic acid) coupling with azidophenyl groups as a photocrosslinker unit. The copolymer spread on PS surface is only covalently grafted at UV irradiated region. Mouse fibroblast STO cells successfully adhere to the copolymer-grafted region at 37°C and detach themselves at a lower temperature. In a similar way, Nash *et al.* [52] have prepared PIPAAm-grafted surface, which is prepared by photo-crosslinkable PIPAAm containing benzophenone unit, and demonstrated a successful 3T3 cell sheet harvest and a graft-polymer-thickness dependency as described above.

### Grafting polymer by ‘grafting-from’ method

Except irradiation procedure, PIPAAm bush can be grafted from the substrate by surface initiated ‘living’ radical polymerizations, including atom transfer radical polymerization (ATRP) and reversible addition-fragmentation chain transfer radical polymerization (RAFT).

Li *et al.* [53] have grafted PIPAAm brush on silicon wafer with a gradient in polymer thickness by ATRP method. A silicon wafer immobilized with ATRP initiator is gradually and vertically immersed into a reaction vessel, while the reaction solution is continuously added to the reaction system. PIPAAm brushes have been grafted on silicon surface with various thickness of PIPAAm chains from 5 to 80 nm. The thickness dependency of cell adhesion/deadhesion is observed on the resultant PIPAAm brushs grafted surface. Human hepatoma (HepG2) cells can only attach and detach themselves from the surface grafted with 20–45-nm thick PIPAAm brushes by changing temperature. Otherwise, HepG2 cells hardly attach to the surface covered with more than 45-nm thick PIPAAm brushes even at 37°C, and the cells barely detached themselves sufficiently from the surface covered with less than 20-nm thick PIPAAm brushes.

Significantly, the density of polymer brushes prepared by ATRP has also been found to affect the characteristic of cell adhesion/deadhesion. Mizutani *et al.* [54] and Nagase *et al.* [55] have found that only polymer brush with a high density and short chain length can control cell adhesion and deadhesion. It is probably because the mobility of polymer chains is relatively restricted and dehydrated of PIPAAm. Decreasing the density of short PIPAAm chain can improve cell attachment at 37°C, but disturb cell detachment at low temperature, probably because the character of hydrophobic basal surface is exposed to the outermost surface of PIPAAm brush. Moreover, increasing the chain length of polymer brush with a higher polymer density disturb cell attachment, probably because the outermost parts of longer polymer chains are induced to be more movable and more hydrated.

The thickness and density dependency of PIPAAm brush on cell adhesion/deadhesion are also found in PIPAAm brush-grafted glass prepared by RAFT [56]. As shown in Table 2, PIPAAm brush-grafted surfaces, which covered with a high polymer density of 0.04 chains/nm^2^ and middle polymer weight (polymer length) (*M*_n_: 49 000 or 23 000), or covered with a middle polymer density of 0.03 chains/nm^2^ and high polymer weight (*M*_n_: 58 000), are found to control BAECs adhesion and deadhesion. Moreover, BAECs are difficult to attach to PIPAAm brush-grafted surface with a high polymer density of 0.04 chains/nm^2^ and high polymer weight (*M*_n_: 58 000). In contrast, attaching BAECs on the surface with a low polymer density of 0.02 chains/nm^2^ are hardly recovered at low temperature.

**Table 2.** The thickness and density dependency of PIPAAm brush on cell adhesion and deadhesion

Density of grafted PIPAAm chains (chains/nm^2^)	Molecular weight of grafted PIPAAm chains (*M*_n_)	Cell adhesive character^a^	
		37°C	20°C
0.02	23 000	Adhesive	Adhesive
	49 000	Adhesive	Adhesive
	58 000	Adhesive	Adhesive
0.03	23 000	Adhesive	Adhesive
	49 000	Adhesive	Adhesive
	58 000	Adhesive	Deadhesive
0.04	23 000	Adhesive	Deadhesive
	49 000	Adhesive	Deadhesive
	58 000	Poor adhesive	Deadhesive

^a^For cell adhesion experiment, BAECs are used.

### Polymer coating method

PIPAAm can be coated on substrate directly by electrostatic interaction. Liao *et al.* [57] have successfully prepared PIPAAm-coated surface by assembling two different PIPAAm copolymers with layer-by-layer technology. PIPAAm copolymers with cationic allylamine hydrochloride segment and with anionic styrene sulfonic acid segment are alternately coated on a glass coverslip surface. Bone marrow-derived human mesenchymal stem cells (hMSCs) are found to adhere more efficiently to and proliferate on the polymeric multilayered surfaces with terminally cationic copolymer coated than only fetal bovine serum (FBS) coated glass coverslip surfaces, because the cationic moiety probably promotes (FN) adsorption as well as cell adhesion in comparison with the anionic moiety. hMSC sheet can be harvested by reducing temperature. Significantly, hMSC in harvested hMSC sheet shows a higher colony forming unit than that cultured on FBS-coated glass surface and harvested by trypsin treatment, which suggest undamaged cell surface proteins on harvested hMSC sheet probably preserve the stemness in the primitive stage and the maintenance of their multi-linage potential *in vitro*. Similarly, Schmidt *et al.* [58] have proposed PIPAAm coating surface by electrostatically coating anionic PIPAAm micro gel on cationic glass surface. L929 mouse fibroblast cells are found to adhere and spread on the PIPAAm micro gel surface at 37°C, and detach from the surface by reducing temperature to 25°C.

Physically polymer coating is another method for preparing the PIPAAm-grafted surface. Loh *et al.* [59, 60] have used triblock copolymer for preparing the PIPAAm-deposited TCPS containing hydrophobic poly[(R)-3-hydroxybutyrate] (PHB) sandwiched between two hydrophilic PIPAAm segments (PIPAAm-PHB-PIPAAm). Molecular dynamics simulation suggests that the copolymer molecules dissolved in organic solvent and assembled themselves to form micelle. When the micelle deposited on hydrophobic surface, the hydrophobic cores of the copolymers in the micelle aggregated and anchored to the hydrophobic surfaces through hydrophobic interaction. As a result, hydrophilic PIPAAm segments are physically coated on and covered the hydrophobic surfaces. Further study demonstrates that more than 90% of coated polymers can remain on the surfaces after being washed with water. As observed in PIPAAm-grafted surface prepared by irradiation method and ‘grafting-from’ method, the characteristics of cell adhesion and deadhesion of the triblock copolymer-coated surface is dependent on both the polymer graft density and thickness. The optimal amount and thickness of coated copolymer are 0.566 µg/cm^2^ and 4–8 nm, respectively. MC3T3-E1 osteoblast and hMSCs can attach and detach from the optimized copolymer-coated surface by reducing temperature. Similarly, Nakayama *et al.* [61] have synthesized poly(butyl methacrylate-*block-N*-isopropylacrylamide) (PBMA-*b*-PIPAAm) by RAFT and coated the block polymer on PS surface by spin coating method. As described above, the hydrophobic segment of PBMA deposits on hydrophobic PS surface to obtain PIPAAm-grafted surface. Polymer density and thickness are optimized to be 1.44 µg/cm^2^ and 15.4 nm for BAECs adhesion and deadhesion, respectively. Moreover, BAEC sheet has successfully recovered from the PBMA-*b*-PIPAAm-coated surface.

Recently, Sakuma *et al.* [62] have produced PIPAAm-grafted surface by Langmuir–Schaefer (LS) method. Dodecyl terminated-PIPAAm (PIPAAm-C12) prepared by RAFT was dropped on an air–water interface and formed Langmuir film by compressing. The Langmuir film has been transferred on cover-glass substrate to produce PIPAAm-C12 transferred surface (PIPAAm-LS surface). Adhesion and detachment of BAEC is observed from the PIPAAm-LS surfaces. BAECs adhere on all of PIPAAm-LS surfaces similarly at 37°C, however, attaching BAECs detach themselves from the surface with a high density and short chain of PIPAAm-C12 molecule more rapidly at 20°C.

## Improvement of Temperature-Responsive Cell Culture Surface for Functional Cell Sheet Culture and Recover

### Fabricating biomolecule on PIPAAm-grafted surface

Bioactive molecules, such as cell adhesive peptide [63, 64], antibody [65] and cell growth factor [66], are attempted to be introduced into PIPAAm layer to enhance the biocompatible and bioactivity of temperature-responsive cell culture surfaces. However, biomolecules are unable to be modified on PIPAAm layer directly, since the immobilization procedure mentioned above damage the biomolecules themselves and/or their structures. Reactive moiety such as acrylic acid has been used to conjugate various biomolecules on PIPAAm layer. However, introducing of carboxylate group increases the phase transition temperature of resultant copolymers, and dampens the sharp phase transition of copolymers, resulting in poor cell adhesion. To overcome these shortcomings, Aoyagi *et al.* [67] have developed a functional monomer, 2-carboxyisopropylacrylamide (CIPAAm), to introduce a biomolecule. This monomer has a side chain structure similar to that of *N*-isopropylacrylamide (IPAAm). As a result, P(IPAAm-*co*-CIPAAm) shows a sharp phase transition in response to temperature similar to PIPAAm. Significantly, biomolecules can be immobilized on PIPAAm layer through the reactive carboxylate group of CIPAAm, and keep their bioactivity.

Arg-Gly-Asp-Ser peptide (RGDS), which exists in type I collagen, FN and other ECM proteins, promoting cell adhesion [68], has been modified on the P(IPAAm-*co*-CIPAAm)-grafted surface via the functional groups of CIPAAm [63]. The resultant surface has been found to promote the adhesion and spreading of human umbilical vein endothelial cells (HUVECs) via affinity interactions between the immobilized RGDS on substrate and the integrin receptor on cells with serum-free medium at 37°C, whereas few cells spread even on TCPS. Moreover, the adhering cells can detach spontaneously to form a cell sheet at 20°C, due to the extension of hydrated polymer chains. Furthermore, more biomolecules can be immobilized on PIPAAm layer via affinity binding between biomolecules themselves. Biotinylated RGDS peptides are coupled onto the streptavidin-modified PIPAAm layer without coupling reagents [64]. Similar as the surface immobilized directly with RGDS peptide, both serum-free cell culture and trypsin-free cell recovery can be achieved with the resultant surface. Recently, heparin, which can combine with various heparin-binding proteins, has been introduced in P(IPAAm-*co*-CIPAAm)-grafted surface by Arisaka *et al.* [66]. It is well known that serum in cell culture medium consists of several kinds of heparin-binding proteins such as growth factors and bioactive proteins. Thus, introducing heparin in PIPAAm layer can promote the concentration of the growth factors and bioactive proteins on cell culture surface, resulting in an acceleration of cell proliferation. The rate of adhesion and growth of NIH/3T3 cells on TCPS and PIPAAm-grafted surfaces are similar, where the doubling times (*T*_d_) are both 24.9 h. In contrast, the heparin-immobilized surface enhanced cell adhesion and proliferation (*T*_d_: 20.4 h). Furthermore, basic fibroblast growth factor (bFGF), which is also a kind of heparin-binding protein, is bound on heparin-immobilized surface before cell culture. The bFGF/heparin-immobilized surface further accelerates cell adhesion and proliferation (20 ng/cm^2^ bFGF is bound on heparin-immobilized surface) (*T*_d_: 18.8 h). The fabrication of NIH/3T3 cell sheet on bFGF/heparin-immobilized surface is reduced to 3 days, which is faster than that on PIPAAm-grafted surface (5 days).

### Micropatterned temperature-responsive cell culture surface

The suitable function and survival of tissues and organs are strongly depended on heterogeneous cell environment and complicated cell structure [69–71]. To construct a biomimetic tissue *in vitro*, cellular micropatterning technology has been developed as a powerful tool to manipulate cell positioning and morphology. Combining with the micropatterning technology, several tissue-like constructs have been fabricated on temperature-responsive cell culture surface.

Tsuda *et al.* [72] have developed a complex heterogeneous cell culture system by using a patterned temperature-responsive cell culture surface, where two types of temperature-responsive polymer patterned on the surface by EB irradiation with a stainless mask, namely PIPAAm and *n*-butyl methacrylate (BMA)-*co*-grafted PIPAAm, exhibit different LCST for cell adhesion/deadhesion. Cells can selectively attach to polymer-grafted surface by controlling the culture temperature. At 27°C, rat primary hepatocytes (RPHCs) are seeded on the surface and attach to P(IPAAm-BMA) regions (∼1 mm in diameter) selectively, where P(IPAAm-BMA) chains is dehydrated and hydrophobic. However, RPHCs fail to attach to neighboring PIPAAm regions, where PIPAAm chains are hydrated and hydrophilic at 27°C. Sequentially, after increasing culture temperature to 37°C, BAECs are seeded on the surface and exclusively attach to hydrophobic PIPAAm domains, resulting in patterned co-culture of RPHCs and BAECs. The immunofluorescent studies have demonstrated the albumin production of RPHCs, suggesting that RPHCs are reserved during the low temperature culture at 27°C. The co-culture system is found to assist in the functional survival of hepatocytes, which is important for liver regeneration. Moreover, by reducing temperature to 20°C, since both P(IPAAm-BMA) and PIPAAm regions become hydrophilic, a patterned cell sheet containing both RPHCs and BAECs can be recovered with heterotypic cell interactions.

**Figure 2.**
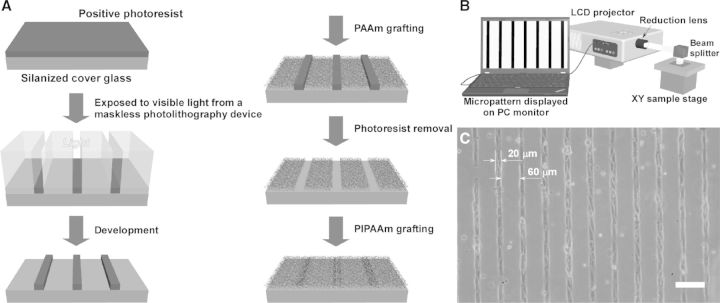
(**A**) Schematic illustration of the fabricating a micropatterned temperature-responsive cell culture surface. (**B**) Schematic illustration of the modified LCD projector for maskless photolithography. (**C**) Phase contrast microscopic photographs of human umbilical vein endothelial cells HUVECs on 20 µm-wide adhesive PIPAAm-grafted domains at 37°C. Scale bar: 100 µm. (Reprinted with permission from Ref. [75] © 2007 Elsevier.)

Many relevant functions of cell depend critically on the organizational structure of cells and ECM [73, 74]. By fabricating cell non-adhesion regions with hydrophilic polymer, a stripe micropatterned temperature-responsive cell culture surface has been prepared to create a blood capillary-like network for 3D thick tissue [75]. A modified commercially available liquid crystal display (LCD) has been used to fabricate the micropatterned surfaces [76, 77]. This new maskless photolithography technique avoids the requirement of photomask, and produces patterns as well as show in PC monitor on photoresist-coated surface through the LCD projector with reduction lens (Fig. 2B). As shown in Fig. 2A, polyacrylamide (PAAm) is grafted on the light-exposed areas, and PIPAAm is grafted on the residual areas after removing the photoresist. HUVECs selectively adhere onto 20 μm-wide micropatterned PIPAAm domains, and not onto 60 μm-wide PAAm domains due to the hydrophilic property at 37°C (Fig. 2C). Moreover, HUVECs on PIPAAm domains extend themselves, and aligned with their long axis to be parallel to the micropattern. By using a cell sheet stacking manipulator, micropatterned HUVEC sheet is harvested and sandwiched between neonatal normal human dermal fibroblasts (NHDFs) sheets to fabricate a 3D cell construct. HUVECs in the multi-layered tissue are found to maintain their elongated structures and self-organize into capillary-like networks after a 5-day culture. Further studies demonstrate that the networks of ECs can promote the formation of microvasculature inside the multilayered cell sheets and can anastomose to the host vessels after transplantation *in vivo*.

**Figure 3.**
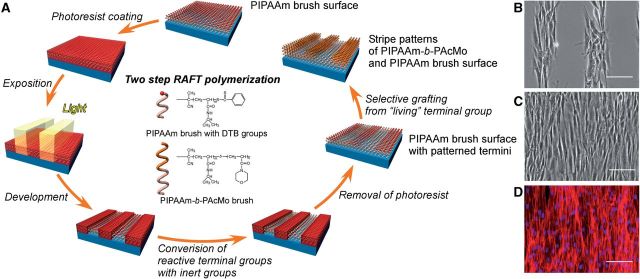
(**A**) Schematic illustration of the fabrication of micropatterned temperature-responsive PIPAAm/PIPAAm-*b*-PAcMo patterned brush surface via a two-step RAFT polymerization. PAcMo have been grafted from the terminal of PIPAAm chains via reactive DTB groups. (**B**) Phase contrast image shows that NHDFs only adhere on 100 µm-wide PIPAAm regions of PIPAAm/PIPAAm-*b*-PAcMo patterned surface after 24 h culture. (**C**) Phase contrast image and (**D**) fluorescence image show that NHDF sheet is formed on a 50-µm-wide PIPAAm/PIPAAm-*b*-PAcMo patterned surface after a 5-day culture. (D) Actin and nuclei of NHDFs are stained with AlexaFluor568-phalloidin (red) and Hoechst 33258 (blue), respectively. Scale bars: 100 µm. (Reprinted with permission from Ref. [78] © 2011 the American Chemical Society.)

Recently, combining the surface-initiated RAFT polymerization with the maskless photolithography technique, Takahashi *et al.* [78] have developed a micropatterned PIPAAm-grafted surface to fabricate a well organizational cell sheet. As a ‘living’ radial polymerization method, there is a reactive group at the terminal of grafted polymer brush prepared with RAFT, which can be attempted to graft another polymer from the end of firstly grafted polymer chain. As shown in Fig. 3A, after photoresist coating and its micropatterned exposure, reactive dithiobenzoate (DTB) groups at the end of grafted PIPAAm chains are partially replaced by inactive maleimide groups. The resultant PIPAAm chains with maleimide groups have been avoided further polymerization and formed the PIPAAm domains on the substrate. Subsequently, after removing the residual photoresists from the substrate, PIPAAm chains with reactive DTB groups are grafted with poly(*N*-acryloylmorpholine) (PAcMo), which form block polymer (PIPAAm-*b*-PAcMo) brush domains on the substrate. By the two-step RAFT polymerization, a stripe microtextured polymer brush-grafted surface has been prepared. NHDFs are cultured on the microtextured PIPAAm brush-grafted glass. After a 24-h incubation, NHDFs are confirmed to attach to the PIPAAm brush segments of microtextured surface, and the stripes of attaching cells with a well-organized structure are found (Fig. 3B). Moreover, cells can also migrate to and proliferate on PIPAAm-*b*-PAcMo domains in further incubation, and keep the organized structure. As shown in Fig. 3C, after a 5-day incubation, a well-organized NHDFs sheet is fabricated on the 50 µm-wide PIPAAm/PIPAAm-*b*-PAcMo patterned surface, as well as the alignment of NHDFs is preserved. The fluorescence photograph also shows the orientation of actin filaments in the cell layers, suggesting that the alignments of cytoskeleton is also regulated by the designed microtextured of polymer regions (Fig. 3D). Significantly, decreasing temperature to 20°C, NHDF sheet detached from the microtextured surface shows a different shrinking rate of cell sheet in vertical and parallel directions of cell alignment. (The aspect ratio of vertical/parallel is ∼3.) These results indicate that the well-controlled orientational structure of cells can be reserved after the recovering of cell sheet, which strongly affect the mechanical and biological characteristics of cell sheet.

### Rapid recovery of cell sheets

Cells can detach from the temperature-responsive cell culture surface with their deposited ECM by reducing temperature to 20°C, however, the detachment speed strongly depends on the kinds of cells. Recently, Tang *et al.* [79] have developed a microfluidics device to study the interaction between cells and hydrated PIPAAm layer, where the different detachment profiles have been observed between NIH/3T3 cells and BAECs. NIH/3T3 cells have been found to shrink themselves more quickly than BAECs on hydrated PIPAAm layer, when decreasing the temperature to 20°C. In contrast, shrunk BAECs can be removed from the hydrated PIPAAm layer more quickly than shrunk NIH/3T3 cells. The detachment profiles of NIH/3T3 cells and BAECs have quantitatively been estimated by using a peeling model [79].

**Figure 4.**
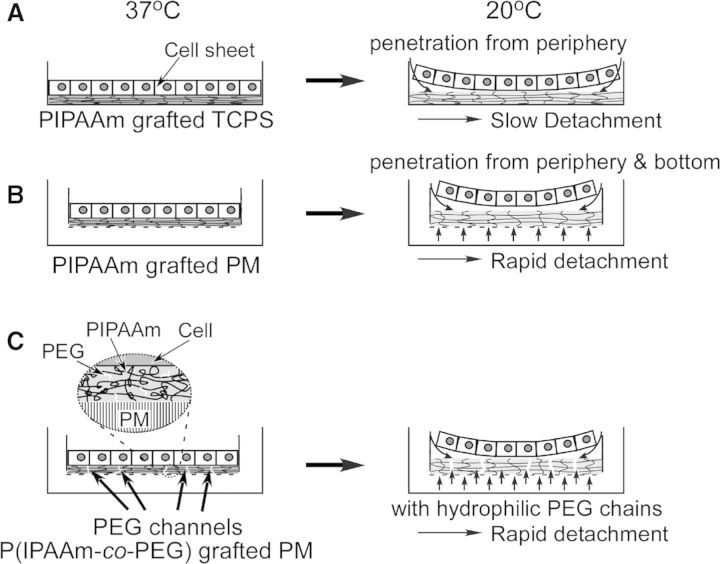
Schematic illustration of water penetration of (**A**) PIPAAm-grafted TCPS, (**B**) PIPAAm-grafted PM and (**C**) P(IPAAm-*co*-PEG)-grafted PM during cell sheet detachment.

Moreover, hydrating process of PIPAAm layer can also affect the detachment of cell sheet. For a general produced PIPAAm-grafted TCPS, the hydration of grafted PIPAAm chains is gradually extended from the periphery of dish toward the central part (Fig. 4A). Thus, recovering a continuous cell sheet from PIPAAm-grafted TCPS requires a long incubation period at a lower temperature. However, the rapid recovery of cell sheet contributes to preserve the bio function and viability of recovered cells, which is important for the rapid fabrication of tissue-like grafts with cell sheet, and reducing the patient burden in clinical treatment.

To accelerate the harvesting procedure of cell sheet, a number of approaches have been developed by physically modifying the surface and/or chemically adapting the structure of PIPAAm chains. Kwon *et al.* [80] have grafted PIPAAm on porous membrane (PM) by EB irradiation to enhance the water pervasion (Fig. 4B). Compared with control surface of TCPS, water molecules penetrate from not only the periphery of dish but also the pore of PM under the attaching cell sheet, which significantly promotes the hydration of PIPAAm layer resulting in rapid recovery of cell sheet. Furthermore, hydrophilic polymer, such as poly(ethylene glycol) (PEG), has also been introduced into PIPAAm layer by co-polymerizating IPAAm and PEG macromonomers to promote the hydration of PIPAAm (Fig. 4C) [81]. Further investigation demonstrates that EC sheet can be harvested from the PIPAAm-*co*-PEG-grafted PM more quickly than that on PIPAAm-grafted PM. These results indicate the grafted hydrophilic PEG chains, which have flexible terminals and form a number of channels for water diffusion, enhance the hydration of PIPAAm layer.

**Figure 5.**
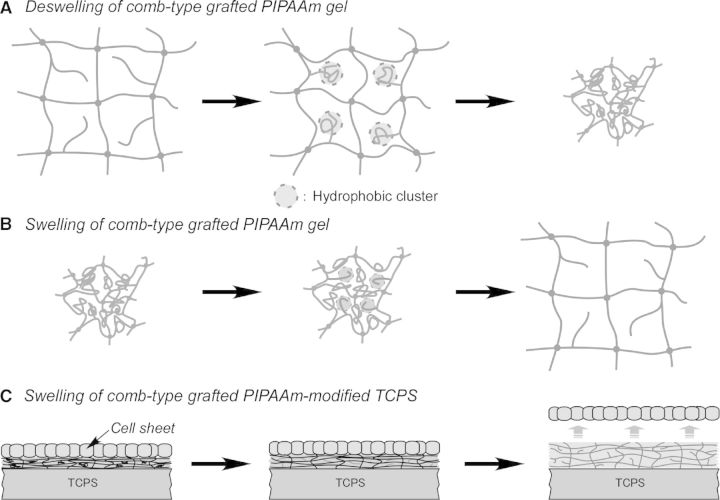
Schematic illustration of (**A**) deswelling and (**B**) swelling of comb-type-grafted PIPAAm gel and (**C**) the swelling of cell culture surface grafting with comb-type-grafted PIPAAm. (Reprinted with permission from Ref. [83] © 2010 Elsevier.)

In previews studies, Yoshida *et al.* have proposed a comb-type-grafted PIPAAm (ctPIPAAm) hydrogel, which contains PIPAAm graft chains with freely flexible terminals. Significantly, the terminal-free-grafted PIPAAm chains show a unique characteristic enhancing the swelling/de-swelling rate of the PIPAAm hydrogel in response to temperature change [82, 83]. As shown in Fig. 5A, above LCST of PIPAAm, the grafted PIPAAm chains with freely flexible terminals dehydrate and aggregate faster than conventional cross-linked PIPAAm chains in gel network. The ctPIPAAm gel can shrink more quickly, because PIPAAm chains with freely flexible terminals form hydrophobic clusters inside the polymer network and water molecules can rapidly be excluded from the gel before the formation of skin layer [82]. In contrast, below LCST, the grafted PIPAAm chains are hydrated faster, resulting in the rapid hydration and swelling of ctPIPAAm gel (Fig. 5B). Tang *et al.* [84] have newly developed a ctPIPAAm modified surface for providing a rapid cell sheet recovery (Fig. 5C). Similarly, PIPAAm macromonomer is mixed with IPAAm monomers and incorporated into PIPAAm-grafted surface by EB irradiation, resulting in a ctPIPAAm gel grafting on TCPS (ctPIPAAm-grafted TCPS). At 37°C, BAECs can adhere well and grow to confluence on ctPIPAAm-grafted TCPS as well as PIPAAm-grafted TCPS. Reducing temperature to 20°C, BAEC sheets can be harvested from conventional PIPAAm-grafted TCPS in 55 min. In contrast, BAEC sheet can be harvested within 25 min from ctPIPAAm-grafted TCPS with 5.0 wt% macromonomers in feed. These results suggest that the resultant surfaces not only keep the dehydration of PIPAAm layer without influence of hydrophilic units such as PEG, but achieve a rapid hydration of PIPAAm network due to the freely mobile end of graft PIPAAm chains, resulting in a rapid recovery of cell sheet.

## Fabrication of Functional 3D Tissues with Cell Sheet

### Cell sheet manipulation technology

**Figure 6.**
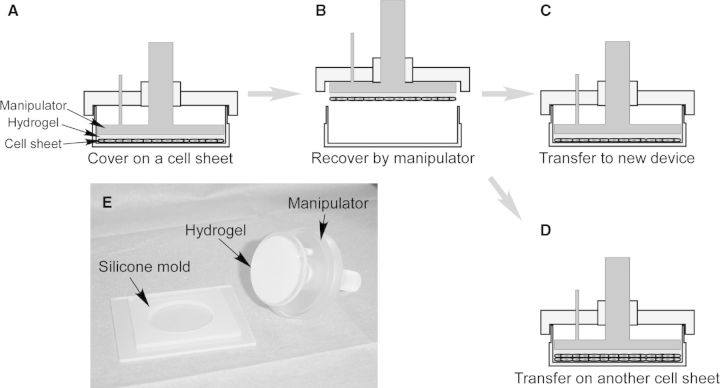
Schematic illustration of the cell sheet manipulator for transferring cell sheet and stacking cell sheet. (**A**) Cell sheet has been covered with a hydrogel-coated manipulator and incubated for 20 min at 20°C. (**B**) Cell sheet has been recovered by the manipulator. (**C**) The harvested cell sheet has been transferred to a new cell culture dish. (**D**) The recovered cell sheet has been stacked on another cell sheet. (**E**) Macroscopic view of a cell sheet manipulation device.

Fabricating a 3D cell-dense thick tissue retaining the functions of cells/tissues *in vitro* is important for tissue engineering and regenerative medicine. Without enzymatic treatment in cell recovery, cell sheets harvested by temperature change completely maintain the cell–cell junctions, cell surface proteins and ECM, which makes it possible to fabricate 3D tissues without scaffold by layering the cell sheets. Although various cell sheets can be harvested from a number of temperature-responsive cell culture substrates mentioned above, detached cell sheets usually shrink and fold themselves during the detachment procedure. To recover a flat and spread cell sheet and fabricating 3D tissue-like constructs, a cell sheet manipulator has been developed [85]. As shown in Fig. 6, a cell sheet manipulation device, consists of a silicone rubber mold, a supporting/recovering hydrogel and a stamp-like manipulator, has been developed to recover a cell sheet from the temperature-responsive cell culture dish. Either fibrin or gelatin gel has been fabricated at an optimum concentration and time in the silicone mold, which has been used as a supporting/recovering material to attach to cell sheet. Cell sheet manipulator with hydrogel has been covered on the cell sheet and incubated for 20 min at 20°C (Fig. 6A). Cell sheet can easily be harvested from the dish by lifting the manipulator, because either hydrogel can tightly adhere onto the surfaces of cells (Fig. 6B). By using the cell sheet manipulator, cell sheet can be recovered without shrinking and folding. The recovered cell sheet adhered on the supporting hydrogel is thinner and has a larger surface area than cell sheet detaching spontaneously. Moreover, with the manipulator, the recovered cell sheet can be shifted to another cell culture dish, and ECM deposited in the basal surface of cell sheet can attach to the new substrate (Fig. 6C). 3D tissue can be constructed by stacking cell sheets repeatedly. The layered cell sheets adhere to each other via ECM (Fig. 6D). The recovered cell sheet adhered on the hydrogel can be easily manipulated and transplanted. With this simple device, 3D cardiac tissues have been fabricated by stacking cardiac cell sheets, which can pulsate spontaneously, synchronously and macroscopically [86, 87]. By layering different cell sheets, 3D tissue with capillary-like prevascular networks can also be construed as mentioned above. The layered cell sheets provide stronger tissue functions, stronger regeneration functions and stronger therapeutic effects than a single cell sheet. Recently, for the future commercial and clinical applications, an automatic cell sheet stacking apparatus has been developed by combining the cell sheet manipulator technique and the industrial robot technology [88]. Similar as mentioned above, gelatin hydrogel has been fixed on the bottom of manipulator as the supporting material for adhering cell sheets. By optimizing the production conditions, such as cell seeding density, manipulator weight and stacking times, the apparatus can successfully recover five-layer human skeletal muscle myoblast (HSMM) sheets. In addition, to overcome the weakness of biological hydrogel, such as the strong interaction between cell sheet and biogel and the inflammation reaction during the gel degradation *in vivo*, some synthetic materials such as hydrophilic modified poly(vinylidene difluoride) membrane and polyelectrolyte complexes have also been developed as the support membrane of cell sheet [89, 90].

### *In vitro* fabrication of thick functional 3D tissues via the engineering of vasculature

Cell sheets can be stacked layer by layer with both manual and automatic approach, however, resultant thickness of multilayered cell sheets is not able to be increased effectively, whatever being cultured *in vivo* or *in vitro*. In previous study, neonatal rat cardiomyocyte sheets over three layers do not grow thicker 1 month after being implanted subcutaneously [91]. The thickness of layered human endometrial-derived mesenchymal cell sheets is also limited approximately 40 mm, when the sheets are transplanted and cultured on a dish [92]. Although HSMM sheets can be recovered to fabricate multilayered constructs by the automatic cell sheet stacking apparatus mentioned above, the thickness of five-layer sheets is limited in approximately 70–80 mm, which is found to significantly decrease after 5-day incubation *in vitro* [88]. Further investigation demonstrates that the thickness of engineered tissue is limited by diffusion issues. Namely, oxygen and nutrient supply, and waste removal is not able to be performed among layered cell sheets and/or between the implanted construct of cell sheets and host tissue due to a lack of vasculature, resulting in necrosis inside of the cell sheets.

To overcome thickness limitation for layered cardiac cell sheets and fabricate a functional 3D cardiac tissue with perfusable blood vessels *in vitro*, Sekine *et al.* [93] have prepared a vascular bed for cell sheet culture, which is made from rat femoral muscle and contain a connectable artery and vein. This vascular bed is placed in a custom-made tissue culture chamber, which is connected with a microprocessor-controlled delivery pump, a pH probe, a dissolved oxygen probe, a pH transmitter, flow transmitter, a pressure transmitter, CO_2_ gas flow controllers and a data acquisition system to constitute a one-pass bioreactor. Three-layer cardiac cell sheets construct cocultured with endothelial cells have been transplanted on the vascular bed, as well as the bioreactor is perfused with culture media. After 3-day culture, endothelial cells have been found to form tubular lumens and connect to capillaries in the vascular bed, resulting in perfusable blood vessels in the cardiac cell sheets *in vitro*, while the cardiac construct shows simultaneous beating. Thicker tissues can be prepared by repeatedly transplanting additional three-layer cell sheets, as well as the perfusable blood vessels anastomose completely and connect to the vascular bed. 12-layer cell sheets construct have been produced successfully by overlaying three-layer cell sheets four times on the vascular bed.

**Figure 7.**
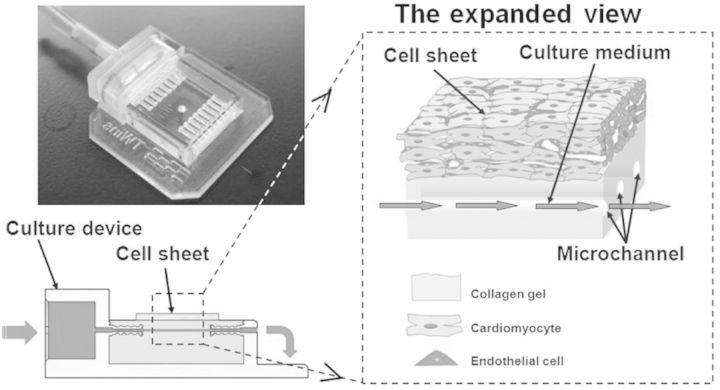
Schematic illustration of the cell sheet culture device with tissue surrogates. The left picture shows a photograph of the culture device.

Sakaguchi *et al.* [94] have developed a new approach, where tissue surrogates have been used as cell sheets culture bases to fabricate the functional 3D cardiac tissues with perfusable vasculature. A collagen gel patch containing several parallel 300 µm-diameter channels inside replace the vascular bed, which is prepared in a culture device made by 3D printer (Fig. 7). The culture device is connected with a microsyringe pump, O_2_ and pH sensors, a pressure sensor, CO_2_ gas flow controllers and a data acquisition system to form a circular bioreactor. The collagen gel imitates the subcutaneous ECM, as well as the microchannels imitate the vasculature. When the cardiac cell sheets mixed with endothelial cells are cultured on the collagen gel patch, culture medium can diffuse from the microchannels into the gel and supply oxygen and nutrients to cell sheets. Significantly, endothelial cells are found to migrate into collagen gel, and form vascular networks connected to microchannels, when the cell sheets are cultured with the medium combining with vascular endothelial growth factor and bFGF. Similarly as being cultured on the vascular bed, a vascularized thick tissue can be constructed by layering the three-layer cell sheets repeatedly. These bioreactors mentioned above provide a new approach to fabricate the 3D thick tissue with cell sheets *in vitro* for tissue engineering and regeneration medicine.

## Application of Cell Sheet Engineering for Animal Models and Clinical Studies

Various cells such as corneal epithelial cells [95], oral mucosal epithelial cells [96], renal epithelial cells [97, 98], epidermal keratinocytes [99], periodontal ligament cells [100, 101], chondrocyte [102, 103], middle ear mucosal cells [104], pancreatic islet cells [105–107], hepatocytes [108–110] and thyroid cells [111] have successfully been cultured on temperature-responsive cell culture surface and formed cell sheets. Without enzyme treatment, the harvested cell sheets preserve the cell–cell junctions and deposited ECM. As a result, cell sheets can easily and firmly attach to another cell culture surface or be layered on other cell sheet. Significantly, cell sheet may be transplanted to the host tissue without the need for any sutures. Therefore, cell sheets can be performed as an engineered tissue for various therapeutic treatment in animal models or clinic.

In clinic studies, Nishida *et al.* [95] have firstly reconstructed the corneal surfaces with corneal epithelial cell sheets for the patients, who suffered from severe traumas (thermal or chemical burns) or eye diseases including the Steve-Johnson syndrome and ocular pemphigoid. Recently, for the patients lost bilateral corneal cells, autologous oral mucosal cells, which are closely similar to native corneal epithelium cells, are used to prepare cell sheets for the damaged corneal [95, 112]. The clinic studies performed in Japan and France demonstrate that the transplanted cell sheet is a safe effective engineered tissue-product for reconstructing the healthy ocular surfaces. Ohki *et al.* [113, 114] and Kanai *et al.* [115] have prepared the oral mucosal cell sheets for regenerating esophageal mucosa after esophageal endoscopic submucosal dissection for cutting off superficial esophageal neoplasm. The two-layer oral mucosal cell sheets have been transplanted to ulcer surfaces using an endoscope without suturing to cover the esophageal ulcerations for preventing postoperative inflammation and stenosis. Investigative clinical studies have been performed for examining the protocol as standardized regenerative medicine for esophagus diseases at Karolinska University Hospital (KUH) by the collaboration of Tokyo Women’s Medical University and KUH. Sawa *et al.* [116] have proposed skeletal myoblast sheets transplantation for improving the cardiac performance for dilated cardiomyopathy. Four-layer myoblast sheets have been implanted on five sites (20 myoblast sheets in total) from the anterior to lateral surface of the dilated heart. The cardiac function has been improved remarkably. The patient received the cell sheets therapy discontinue using a left ventricular assist system without any arrhythmia and avoid heart transplantation. Ishikawa and Iwata *et al.* [117] have reported a clinic study for the therapy of periodontal disease by using autologous periodontal ligament cell sheets. Transplantation of layered periodontal ligament-derived cell sheets has been found to induce periodontal regeneration, including bone regeneration, cementum formation and well-oriented collagen fibers.

Transplanting cell sheet for regenerative therapy has several advantages over direct injection cell suspensions or tissue engineering using biodegradable scaffolds: (i) Intact cellular functions and high cell density can be preserved during the recovery procedure and the transplant of cell sheets. In contrast, only a few cells have been located on target tissue in the direct injection of cell suspensions; (2) with the aid of intact deposited ECM, cell sheets can directly be transplanted to the host tissue and even to wound sites without any suture; (3) cell sheets can be implanted to the host tissue without any carrier or support material, which prevent the strong inflammatory responses due to the biodegradation of scaffolds.

## Conclusion

Temperature-responsive cell culture surfaces have been developed by modification of PIPAAm and PIPAAm derivate via various methods. An intact cell sheet can successfully be recovered from the temperature-responsive surface without enzyme treatments. Combining with the cell sheet manipulation technology and bioreactor, a functional 3D tissue-like construct can be fabricated with cell sheets for tissue engineering and regeneration medicine.
